# Efficient dataset extension using generative networks for assessing degree of coating degradation around scribe

**DOI:** 10.3389/frai.2024.1456844

**Published:** 2024-12-13

**Authors:** Dominik Stursa, Pavel Rozsival, Petr Dolezel

**Affiliations:** Faculty of Electrical Engineering and Informatics, University of Pardubice, Pardubice, Czechia

**Keywords:** coil coating, delamination, degradation, semantic segmentation, deep learning, generative adversarial network

## Abstract

A novel methodology for dataset augmentation in the semantic segmentation of coil-coated surface degradation is presented in this study. Deep convolutional generative adversarial networks (DCGAN) are employed to generate synthetic input-target pairs, which closely resemble real-world data, with the goal of expanding an existing dataset. These augmented datasets are used to train two state-of-the-art models, U-net, and DeepLabV3, for the precise detection of degradation areas around scribes. In a series of experiments, it was demonstrated that the introduction of synthetic data improves the models' performance in detecting degradation, especially when the ratio of synthetic to real data is carefully managed. Results indicate that optimal improvements in accuracy and F1-score are achieved when the ratio of synthetic to original data is between 0.2 and 0.5. Moreover, the advantages and limitations of different GAN architectures for dataset expansion are explored, with specific attention to their ability to produce realistic and diverse samples. This work offers a scalable solution to the challenges associated with creating large and diverse annotated datasets for industrial applications of coil coating degradation assessment. The proposed approach provides a significant contribution by improving model generalization and segmentation accuracy while reducing the burden of manual data annotation. These findings have important implications for industries relying on coil coatings, as more efficient and accurate degradation detection methods are enabled.

## 1 Introduction

An established method of coil coating plays a pivotal role in applying organic coatings onto rolled metal strip substrates, as discussed in Jandel (2019) and The National Coil Coating Association (2020). This process aims to achieve a uniform, high-quality, and enduring finish on metal surfaces, catering to diverse applications like building exteriors, metal roofs, wall panels, garage doors, office furniture, vending machines, food service equipment, and more. Notably, coil coating extends its utility to advanced applications, including cool metal roofing materials, smog-eating building panels, antimicrobial products, anti-corrosive metal parts, and solar panels.

The significance of coil coating lies in providing a thin yet robust and flexible protective layer that effectively shields materials against corrosion. Despite its efficacy, this protective layer is susceptible to mechanical damage, such as scribes or scratches, leading to irreversible changes due to exposure to environmental elements, see Bastos and Simões (2009). This damage can manifest in various forms, ranging from chalking and blistering to flaking or rusting of the coated material. Consequently, evaluating the performance of coated surfaces under conditions accurately simulating outdoor exposure becomes imperative.

**Figure 1:**
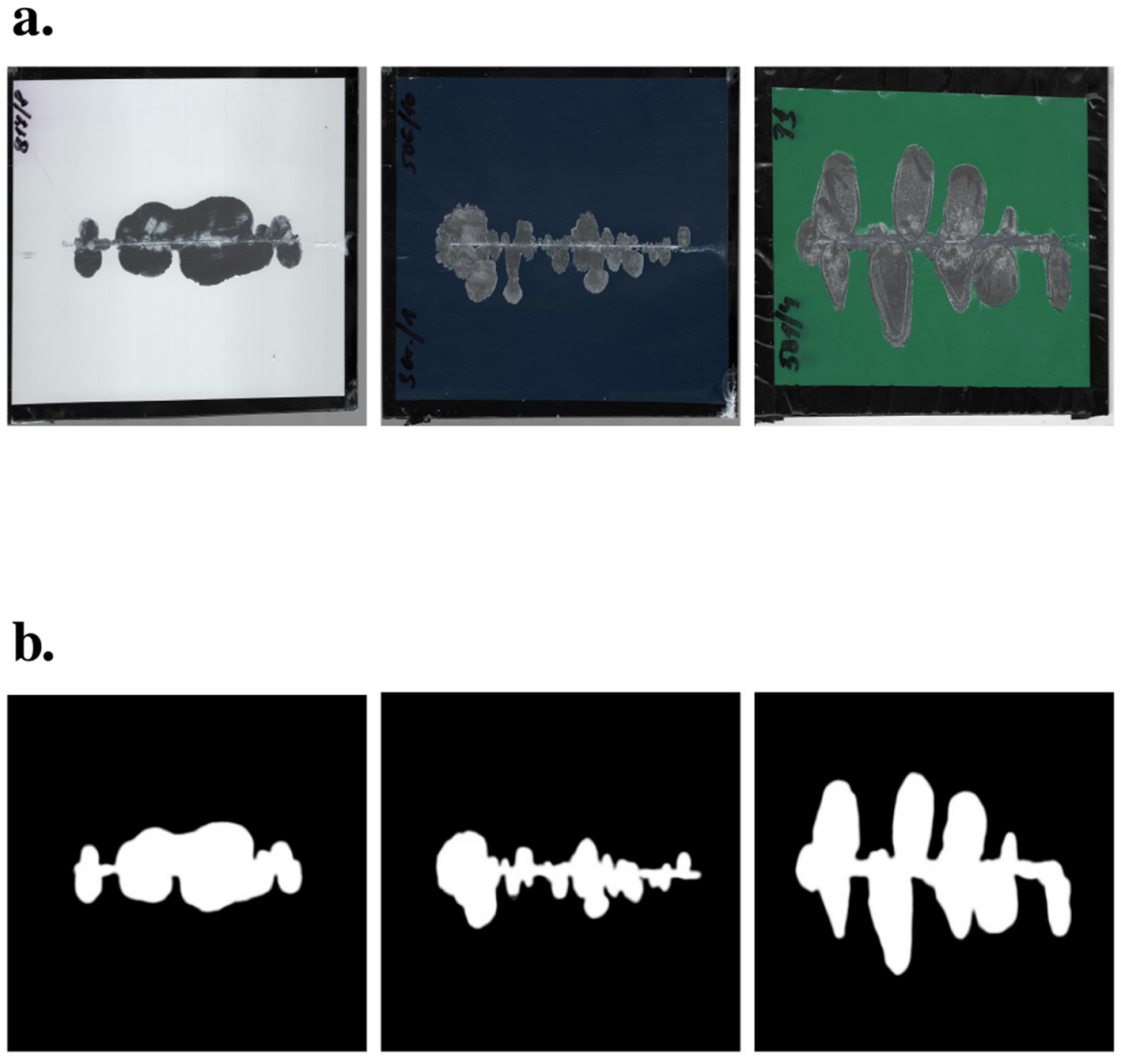
Examples of degraded area determination. **(A)** Test specimen after removal from the salt fog chamber, displaying the visible signs of surface degradation. **(B)** Example of degradation detection, where the degraded area around the scribe is highlighted. The degradation zone is calculated by determining the ratio of the affected area to the total area of the specimen, following the guidelines of ISO 4628-8.

The assessment of degradation resistance of coil-coated materials adheres to the European Standard EN 13523-8, titled “Coil coated metals. Test methods. Resistance to salt spray (fog).” This standard involves subjecting a test specimen treated with coil coating to salt fog at predefined temperature and duration. Subsequently, the specimen undergoes testing following the International Organization for Standardization (ISO) 4,628 standard, titled “Paints and varnishes. Evaluation of degradation of coatings. Designation of the quantity and size of defects and intensity of uniform changes to appearance.” In simpler terms, the objective is to evaluate the extent of surface degradation on the test specimen, as illustrated in Figure 1. The figure demonstrates the process of degradation detection, where a test specimen is first exposed to salt fog (Figure 1A), and the degraded area around the scribe is subsequently identified and measured (Figure 1B). The degree of degradation is determined by calculating the ratio of the affected area to the total area of the specimen, following the ISO 4628-8 standard.

**Figure 2:**
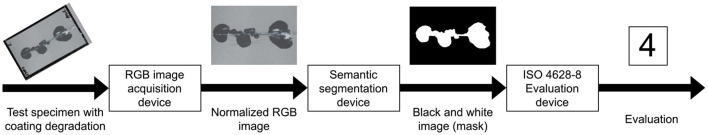
A pipeline of the autonomous method for the assessment of the degree of degradation around a scribe presented in Rozsivalova et al. (2022). First, the test specimen is scanned by an image acquisition device and the image is normalized to a defined resolution. Then, a semantic segmentation tool provides a mask of the image, which represents a degraded segment of the specimen. Finally, the specimen is evaluated using the ISO 4628-8 Standard.

While the manual determination of the degraded area is a common practice, as discussed in Bastos and Simões (2009) and Hanus (2011), there is a scarcity of automatic image processing-based methods. Existing methods include the use of office scanners in combination with commercial software like Adobe Photoshop, as presented in Blanchard et al. (2014). However, these approaches are limited to specific material types, surfaces, or colors. In contrast, industrial practices demand universally applicable methods. Addressing this need, the authors Rozsivalova et al. (2022) proposed a technique based on semantic segmentation using fully-convolutional neural networks—see Figure 2 for an illustration of the technique. The results presented in this work demonstrate the effectiveness of U-shaped fully convolutional networks in automatically detecting the degradation area of surfaces treated with coil coating.

**Figure 3:**
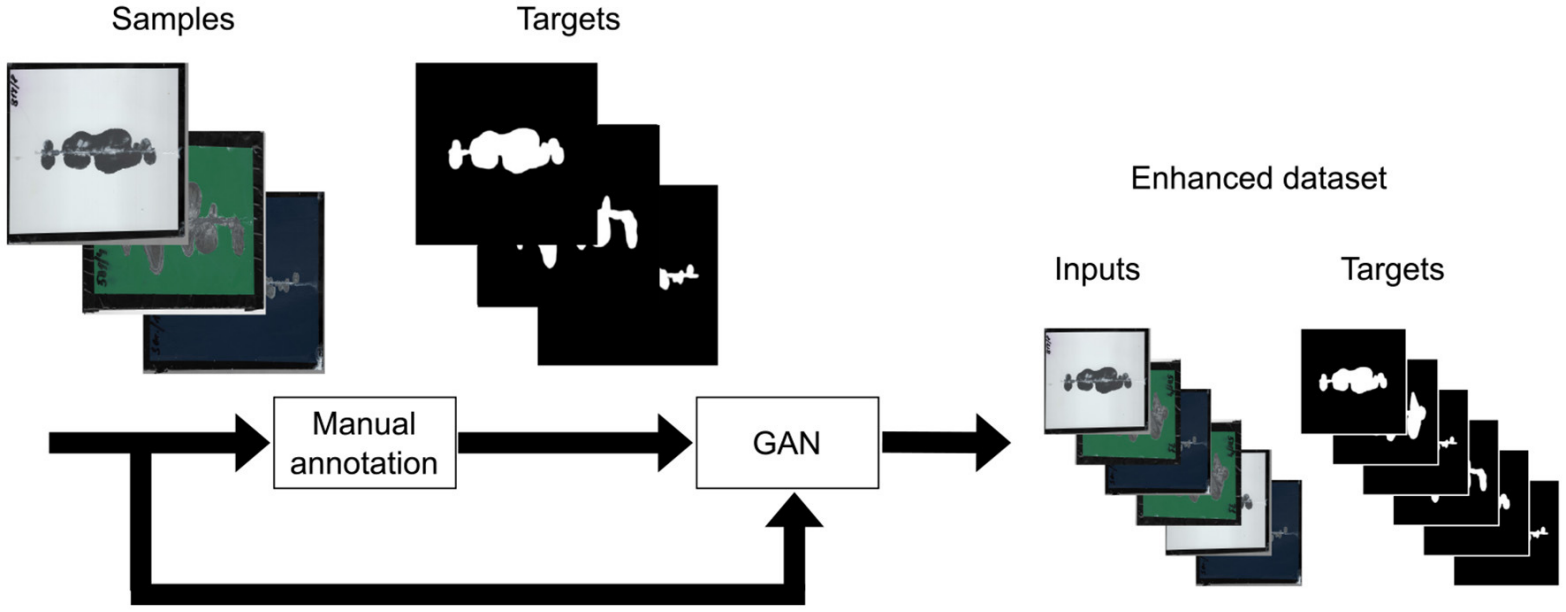
Process of dataset augmentation using a Deep Convolutional Generative Adversarial Network. The original dataset, consisting of manually annotated input images and corresponding target images (masks), is used to train a GAN model. The generator network creates synthetic input-target pairs, simulating degradation patterns on coil-coated surfaces. These synthetic images are evaluated by a discriminator network, which distinguishes between real and generated samples. The final output is an enhanced dataset that combines both real and synthetic data, providing a more diverse and extensive training set for the neural network models tasked with detecting surface degradation. This augmentation method improves the model's ability to generalize and accurately detect degradation in various conditions.

A significant limitation of data-driven methods, particularly those based on semantic segmentation, is the requirement for a large and comprehensive training set covering significant states of the problem. The creation and correct annotation of such a dataset pose substantial challenges. This study focuses on exploring the potential of automatically extending a manually created dataset using Generative Adversarial Networks (GAN). GAN, an unsupervised learning framework, consists of two networks, the generator and the discriminator, competing to produce better results. The generator generates data similar to the training data, while the discriminator differentiates between generated and real data. The study aims to propose a GAN-based technique for automated training set enhancement for a semantic segmentation task. The overall pipeline of the technique is depicted in Figure 3, according to Goodfellow et al. (2014).

One of the primary goals of this research is to develop a method for expanding datasets using GANs, which can create synthetic data that mimics real-world conditions. This synthetic data is then combined with real data to improve the performance of neural networks in detecting surface degradation. Throughout our study, we carefully tested different types of GAN models to determine which architecture produced the most realistic and useful data.

In addition, we focused on finding the right balance between the amount of real and synthetic data to ensure that the neural network models–specifically U-Net and DeepLabV3–performed optimally. These models are used to accurately identify areas of surface damage, and the generated data plays a key role in improving their accuracy. Our experiments explore how different proportions of real and synthetic data affect the models' ability to detect degradation, with the goal of finding the most effective configuration for real-world applications.

The paper is organized as follows: In the Materials and Methods section, we begin by outlining the standard for degradation measurement, ISO 4628-8, which guides the preparation and annotation of the dataset. Following this, we introduce the original dataset used in this study, consisting of coil-coated surfaces, and describe the semantic segmentation models employed to assess the effectiveness of our dataset augmentation approach. A key part of the methodology is the introduction of the GAN-based augmentation process, where both synthetic images and corresponding degradation masks (target images) are generated. This section also includes a description of the different variants of extended datasets used for training and testing, as well as the metrics employed for evaluating the models' performance.

In the Results section, we present a comparative analysis of the models trained on the original dataset vs. those trained on the augmented datasets. The discussion includes the impact of different ratios of synthetic-to-original data, revealing insights into how these ratios affect model performance across various architectures. This section further contextualizes these results by comparing them with existing approaches in the literature, particularly in domains like medical imaging, where GAN-based augmentation is widely used. We also discuss how our method differs from similar GAN-based approaches, such as those that generate original images from masks, and highlight the advantages of our approach, which simplifies the dataset preparation process.

Finally, in the Future Directions and Conclusions sections, we summarize the key findings of this research and propose avenues for future work, such as exploring conditional GANs for more controlled data generation and investigating the applicability of this method in other fields where semantic segmentation is critical.

The main contributions of this study include:

- Development of a GAN-based method for dataset augmentation, specifically designed for the detection of coating degradation around a scribe.
- Systematic analysis of the impact of different ratios of synthetic-to-original data on the performance of state-of-the-art semantic segmentation models.
- Introduction of a methodology that generates not only synthetic images but also their corresponding segmentation masks (target images), significantly reducing the time and effort required for manual data annotation.
- A comparison of our GAN-based approach with existing methods, highlighting its applicability in industrial settings where labeled data is scarce.
- Exploration of future directions for improving the control over the types of generated data and applying this approach to other domains.

A unique feature of our approach is that it generates both the synthetic original images and their corresponding degradation masks in a single step. This removes the need for the time-consuming manual annotation process typically required when augmenting datasets with synthetic data, providing a more efficient and scalable solution for tasks requiring semantic segmentation.

The presented work constitutes an extended version addressing the intricacies originally discussed during the 9th International Conference on Control, Decision, and Information Technologies (Dolezel et al., 2023).

## 2 Related work

The coil coating industry has been the subject of substantial research and development, particularly in addressing challenges related to degradation assessment and surface protection. This section provides a brief review of the existing literature, encompassing both traditional methods and recent advancements.

Early research on the degradation of coil-coated materials primarily relied on manual methods for assessment. Studies such as those by Bastos and Simões (2009) emphasized visual inspection and direct measurement of degraded areas. These manual techniques, while foundational in understanding coating performance, had significant limitations, particularly in terms of subjectivity and dependence on the expertise of the inspector. These limitations have led to a growing interest in developing more objective and automated approaches to degradation evaluation, pushing the field toward more advanced methodologies.

Image processing techniques have since emerged as an alternative to manual inspection. Researchers like Kapsalas et al. (2007) and Cringasu et al. (2017) explored the use of automatic image processing for assessing surface degradation. These methods have demonstrated potential, offering more consistent and repeatable results compared to traditional approaches. One notable method was introduced by Blanchard et al. (2014), where office scanners and commercial software, such as Adobe Photoshop, were employed to detect degradation. However, the applicability of such techniques has often been limited to specific material types, surface characteristics, or color variations, which restricts their generalizability in industrial settings.

The advent of deep learning has opened new opportunities for automating the assessment of coil-coated surfaces. Semantic segmentation, a technique that assigns a class label to each pixel in an image, has been applied in recent research. Fully convolutional networks (FCNs), particularly U-shaped architectures, have been shown to be effective in detecting degraded areas on coated surfaces. The work by Rozsivalova et al. (2022) demonstrated the potential of U-shaped FCNs in this context, marking a significant shift toward automated and objective methods for surface degradation assessment. This transition highlights the increasing relevance of deep learning in addressing the limitations of manual and traditional image processing-based methods.

Despite progress in deep learning-based approaches, data-driven methodologies pose several challenges. The primary difficulty lies in the creation and annotation of large, diverse datasets that are necessary for training robust models. As highlighted by Bastos and Simões (2009), the process of generating and accurately annotating datasets for degradation assessment can be both time-consuming and resource-intensive. This issue is particularly pronounced in industrial applications, where large-scale, high-quality datasets are often not readily available. Consequently, innovative approaches are required to overcome these limitations and ensure the generalization of models to real-world conditions.

To address the challenge of limited datasets, various dataset augmentation techniques have been developed, offering effective solutions for improving model performance in tasks such as semantic segmentation. Among these, Generative Adversarial Networks (GANs) have become a prominent method. Introduced by Goodfellow et al. (2014), GANs are employed to generate synthetic data that closely resembles real-world samples. These networks consist of a generator, which creates new data, and a discriminator, which evaluates whether the data is real or synthetic. This adversarial process has proven to be successful in many domains; however, in the specific case of evaluating coating degradation, there are relatively few practical applications of GANs. One notable example is the work by Tao et al. (2024), which employs a GAN-based approach to generate synthetic original images from provided degradation masks. In this method, a degradation mask is used as input, and the GAN generates the corresponding original, undegraded images. This approach significantly enhances the available dataset by reversing the degradation process, offering a novel solution for improving model training and segmentation performance in the context of coating assessment.

In addition to GAN-based augmentation, several other methods are widely used to enhance datasets. Geometric transformations, such as flipping, rotation, and scaling, are commonly employed to create variations of the original images, helping models become invariant to shifts and rotations, as noted by Islam et al. (2024). Color space transformations, which involve adjusting brightness, contrast, and saturation, further enrich the dataset by simulating different lighting conditions, as outlined in the survey by Shorten and Khoshgoftaar (2019). Kernel filters, such as Gaussian blurring or sharpening, are also used to improve the robustness of the model against image noise and degradation, as explored by Nanni et al. (2021).

More advanced techniques include image mixing, where two or more images are blended to create new synthetic samples, and random erasing, which involves randomly removing parts of an image to force the model to focus on the remaining content, as described by Xu et al. (2023). Another promising approach is feature space augmentation, where transformations are applied in the intermediate layers of the neural network rather than on the raw input images. This method enables more complex augmentations, such as interpolating between feature vectors, as demonstrated by Niu et al. (2023).

Adversarial training has also emerged as a powerful augmentation strategy. In this technique, adversarial examples are generated to mislead the model, forcing it to learn more robust decision boundaries, as discussed by Allen-Zhu and Li (2022). Neural style transfer, where the style of one image is transferred to another, creates visually diverse samples that improve the model's ability to handle real-world variability, as highlighted by Camargo et al. (2024).

The current state of research reflects a clear transition from traditional manual methods to more advanced automated techniques, driven by the integration of deep learning and GANs. This evolution underscores the industry's commitment to developing more accurate, efficient, and universally applicable solutions for degradation detection. Future research efforts should focus on addressing the remaining challenges, such as the creation of diverse, high-quality datasets and the generalization of models to real-world applications. Furthermore, the seamless integration of these innovative technologies into industrial workflows will be crucial for ensuring their practical utility. As the field advances, continued exploration of GAN-based data augmentation and deep learning architectures will play a pivotal role in enhancing the reliability and performance of degradation detection systems.

## 3 Materials and methods

The primary objective of this research is to introduce a methodology for extending the dataset utilizing GAN to evaluate the degree of degradation around a scribe. The dataset originally constructed for this purpose in Rozsivalova et al. (2022) serves as the foundation for our experiments. Subsequently, GAN is employed to enrich this dataset, with a specific focus on generating inputs and targets corresponding to the images within the original dataset (refer to Figure 1 for illustrative examples).

To demonstrate the impact of dataset extension, both the original dataset and its augmented counterpart are utilized to train a neural network specializing in semantic segmentation. In this study, we consider U-net introduced in Ronneberger et al. (2015) and DeepLabV3 proposed in Chen et al. (2017), since both architectures are renowned for their exceptional performance in diverse real-world applications.

The ensuing experiments are designed not only to discern the effectiveness of GAN-based dataset extension but also to investigate the nuanced interplay between original and synthetically generated data in optimizing the performance of the semantic segmentation neural network. Through a comparative analysis of the original and extended datasets, coupled with the utilization of both U-net and DeepLabV3 architectures, our aim was to elucidate the nuances that contribute to achieving superior performance in detecting the degree of degradation on surfaces treated with coil coating.

In order to implement the dataset for semantic segmentation of coil-coated surface degradation, the following tools and methods are presented in this section: First, the ISO 4628-8 standard, which governs the measurement of degradation, is introduced to ensure consistent evaluation across all samples (Section 3.1). Next, the original dataset, consisting of annotated images of coil-coated surfaces, is described as the foundation for further experiments (Section 3.2).

Two semantic segmentation models, U-net and DeepLabV3, are utilized to assess the impact of dataset augmentation on model performance (Section 3.3). The core of the proposed method is the use of a deep convolutional GAN to generate synthetic input-target pairs, which are then combined with the original dataset. Different ratios of synthetic-to-real data are explored to determine the optimal configuration for model training (Section 3.4).

One of the key parts of the methodology is determining the optimal ratio of synthetic-to-original data. In Section 3.5, various ratios are explored to investigate how different proportions affect the model's performance. This analysis is critical in identifying the best balance between real and synthetic data for improving segmentation accuracy.

Finally, standard evaluation metrics, including accuracy, precision, recall, and F1-score, are employed to compare model performance on both original and augmented datasets. This structured approach ensures that the effects of dataset augmentation on segmentation accuracy and model generalization are rigorously analyzed (Section 3.6).

### 3.1 Standard ISO 4628-8

Various examination methodologies for coil-coated metals encompass, among others, the quantification of degradation resistance to salt fog, as stipulated by the EN 13523-8 standard. Within this context, a pivotal metric under consideration involves the evaluation of the degree of degradation around a scribe, a parameter meticulously defined by the ISO 4628-8 standard. The testing protocol entails horizontally scribing the test specimen with a sharp edge, subjecting it to a corrosive salt fog environment. Subsequent to a predefined exposure duration, the specimen undergoes a cleansing with tap water, followed by the removal of water residues through the application of compressed air. Any loosely adhered coating is subsequently eliminated using a blade held at a precise angle. Refer to Figure 1 for a visual representation of specimens prepared following this procedural framework.

In accordance with the provisions delineated in the ISO 4628-8 standard, two distinct variants for assessing the degree of degradation around a scribe are defined.

#### 3.1.1 First variant

The width of the area of degradation has to be measured at a minimum of six points uniformly distributed along the scribe. Subsequently, the arithmetic mean is determined and the resulting value is designated as the mean overall width of the zone of degradation, *d*_1_, in millimeters.

The degree of degradation *d*, in millimeters, can be calculated using the equation


(1) $$\begin{array}{l} d=\text{round}\left(\frac{d_{1}-w}{2}\right), \end{array}$$

where *w* is the width of the original scribe, in millimeters.

#### 3.1.2 Second variant

In this methodology, the area of degradation is explicitly quantified. The standard recommends placing a transparent millimeter-grid paper onto the plate and enumerating the squares aligned with the degradation region. Subsequently, the degree of degradation (*d*), expressed in millimeters, can be computed utilizing the following equation.


(2) $$\begin{array}{l} d=\text{round}\left(\frac{A_{d}-A_{l}}{2l}\right), \end{array}$$

where *A*_*d*_ is the area of degradation, including the area of the scribe (in square millimeters), *A*_*l*_ is the area of the scribe in the evaluated area in square millimeters, and *l* is the length of the scribe in the evaluated area (in millimeters).

### 3.2 Original dataset

In order to formulate a robust data-driven strategy for the automated detection of coating degradation, it is paramount to amass an extensive assortment of varied and meticulously annotated samples that manifest differences in color, surface roughness, and reflectivity. Moreover, these samples should exhibit diverse degrees of degradation. In the initial investigation conducted by Rozsivalova et al. (2022), a comprehensive set of 604 coated samples, each measuring 150 × 100 mm, was fastidiously prepared. These samples encompassed coatings in a spectrum of colors, ranging from black, white, green, and gray to orange, red, brown, blue, dark blue, and yellow, encompassing both fine and coarse (textured) variations.

To unveil the uncoated substrate, a small horizontal scratch, measuring 0.5 mm in width, was intentionally made through the coating of each sample using an iron nail. Subsequently, the samples underwent controlled exposure durations in a salt fog chamber, lasting for intervals of 120 h, 240 h, 480 h, 720 h, and 1,440 h. Following the designated exposure periods, meticulous cleaning procedures were applied, and the samples were subjected to scanning using an office-grade scanner. Finally, each individual sample underwent a manual annotation process to derive the target segmentation image essential for training the area degradation detection model.

Eventually, a subset comprising 128 samples was extracted from this dataset to form an independent test set, leaving a pool of 476 samples actively employed in the subsequent experiments detailed in the following sections.

### 3.3 Semantic segmentation neural models

Semantic segmentation is a technique aimed at assigning each pixel in an input image to a specific class or category, thereby providing semantic context to visual data. This approach proves instrumental for a detailed and interpretable analysis of visual information, enabling discernment of specific objects, structures, or contexts within images. In this study, semantic segmentation process is expected to determine areas of coating degradation with pixel-wise precision. Models designed for semantic segmentation leverage sophisticated neural network architectures, such as U-net or DeepLabV3, capable of effectively capturing and interpreting high-level patterns in image data.

#### 3.3.1 U-net

The U-net architecture, initially devised for biomedical image segmentation, stands as a symmetrical and densely pixel-wise prediction model. Comprising an encoder-decoder network structure, it encompasses a contraction path and an expansion path intricately linked through a succession of convolutional layers. Noteworthy in its design is the incorporation of skip connections, facilitating the concatenation of feature maps from both the contraction and expansion paths. This feature enhances the model's ability to precisely localize features and capture intricate details from the input image. U-net has found wide-ranging applications across various imaging tasks, spanning from the segmentation of brain tumors, see Allah et al. (2023), to cell tracking endeavors, as shown in Yuan et al. (2023).

For the purposes of our study, U-net undergoes modification to accommodate input in the form of a (288 × 288 × 3)px RGB image, generating a (288 × 288 × 1)px black-and-white image at the output.

#### 3.3.2 DeepLabV3

DeepLabV3 represents a state-of-the-art convolutional neural network that excels at capturing intricate contextual information and delivering precise segmentation results.

DeepLabV3's core structure includes an atrous spatial pyramid pooling module, designed to aggregate multi-scale contextual information effectively, see Chen et al. (2017). This module employs dilated convolutions at different rates, allowing the network to capture diverse contextual information while maintaining computational efficiency. The encoder-decoder architecture of DeepLabV3 enables it to refine segmentation predictions with fine-grained details, making it particularly well-suited for our task of detecting coating degradation, as indicated in Kang et al. (2024).

Within the framework of DeepLabV3, a notable feature is its adaptability to various backbone networks, allowing for flexibility in accommodating diverse computational and performance requirements. For the purposes of our study, we conducted experiments with different backbone networks, namely Xception, defined in Chollet (2017) and MobileNetV2, proposed in Sandler et al. (2018), integrated into the DeepLabV3 architecture. The selection of these backbone networks stems from their distinct characteristics and computational efficiency. Xception, known for its exceptional performance and capability to capture complex hierarchical features, serves as a powerful backbone for high-precision tasks. On the other hand, MobileNetV2, recognized for its lightweight structure and computational efficiency, is well-suited for scenarios where computational resources may be constrained without compromising segmentation accuracy significantly.

In the context of our study, the DeepLabV3 architecture undergoes an adaptation to accommodate input in the form of (288 × 288 × 3)px RGB images, producing the corresponding (288 × 288 × 1)px black-and-white segmentation outputs.

### 3.4 Generative adversarial network for dataset extension

The objective of this study is to introduce a GAN-based methodology tailored for the extension of the original dataset, comprising input and target images featuring diverse annotations, encompassing variations in color, asperity, and reflectivity. GANs operate through a dual neural network framework, consisting of a generator and a discriminator. The generator network takes a random noise vector as input and endeavors to produce synthetic images that are perceptually indistinguishable from real ones. Conversely, the discriminator network assesses both real and synthetic images, discerning between authentic and synthetic counterparts.

In the current landscape of research, GANs have emerged as pivotal tools for the automated augmentation of image datasets, aimed at enhancing the performance of neural networks. Various GAN architectures have been developed, each offering specific advantages and applications in the realm of image synthesis.

One widely adopted GAN architecture is the deep convolutional GAN (DCGAN), leveraging deep convolutional layers to generate photorealistic images. This architecture has been a breakthrough for synthesizing high-quality images and has found successful applications in diverse domains, from generating realistic faces to producing artistic works, as summarized in Radford et al. (2016).

Another significant variant is the Conditional GAN, see Mirza and Osindero (2014), allowing the specification of conditions for image generation. This architecture has been applied in tasks where generating data with respect to certain parameters or classes is crucial.

For the purpose of expanding training datasets, researchers have explored the CycleGAN architecture, defined in Zhu et al. (2017). This innovative structure facilitates domain-to-domain translations without the need for paired training data, proving useful, especially in scenarios where obtaining precise pairs of input and target images is challenging.

Advancements in GAN architectures continue, with new variants, such as StyleGAN, discussed in Karras et al. (2021), providing even greater flexibility and control over the generative process.

Given the variety of GAN architectures available, it was essential to carefully choose and customize the most appropriate model for our specific task. In this study, we explored multiple GAN variants, selecting those that could best generate synthetic data resembling real-world degradation patterns. The selection process was supported by a versatile toolset from the GitHub repository (Linder-Norén, 2019), which includes a wide range of GAN architectures and implementation tutorials. This toolset enabled the seamless integration of GANs into our workflow and ensured the correct implementation for generating synthetic data.

The evaluation of the generated data involved a detailed analysis of both the accuracy and visual fidelity of the synthetic images, comparing them to real data. We assessed key characteristics such as color, texture, and the granularity of surface degradation. This thorough evaluation allowed us to understand the strengths and limitations of various GAN models.

**Figure 4:**
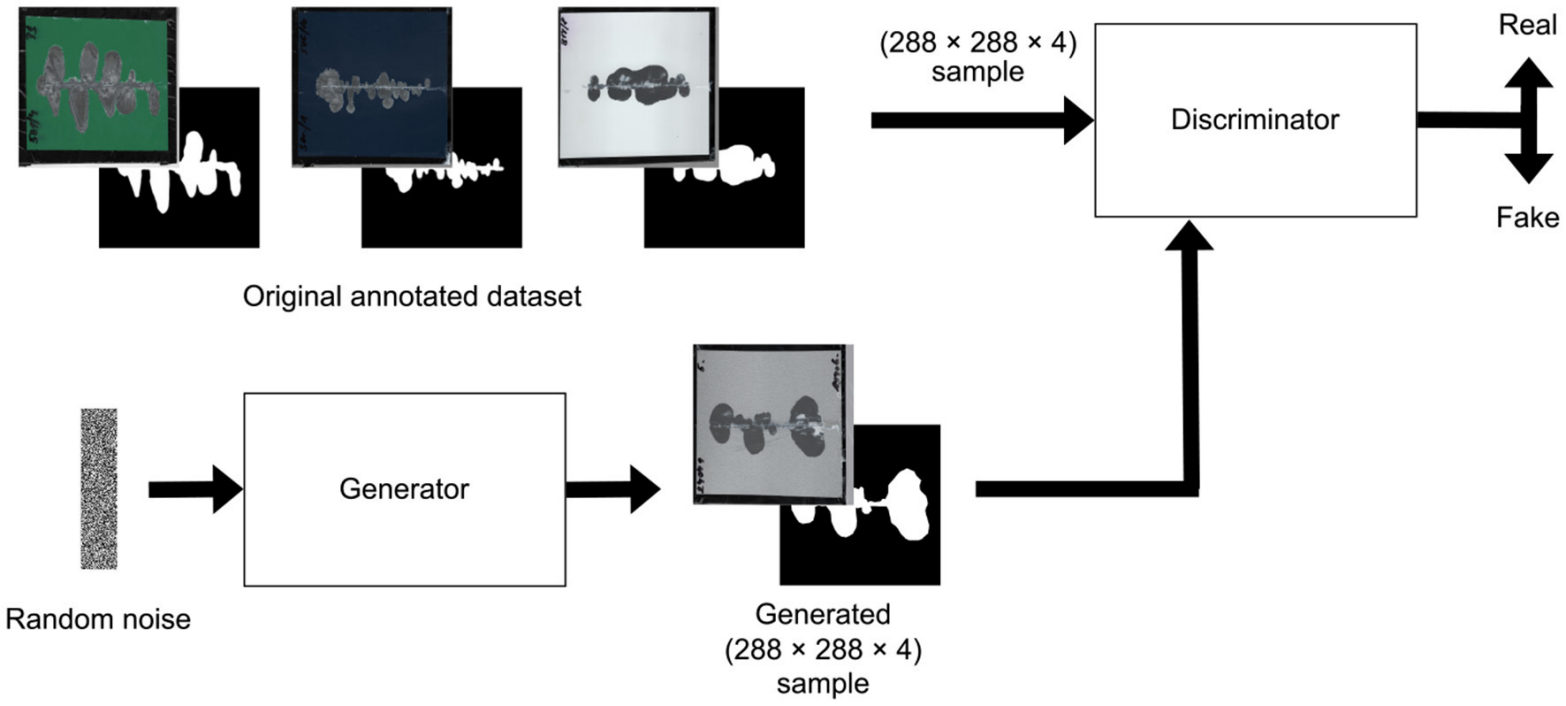
Deep convolutional generative adversarial network for dataset extension.

**Figure 5:**
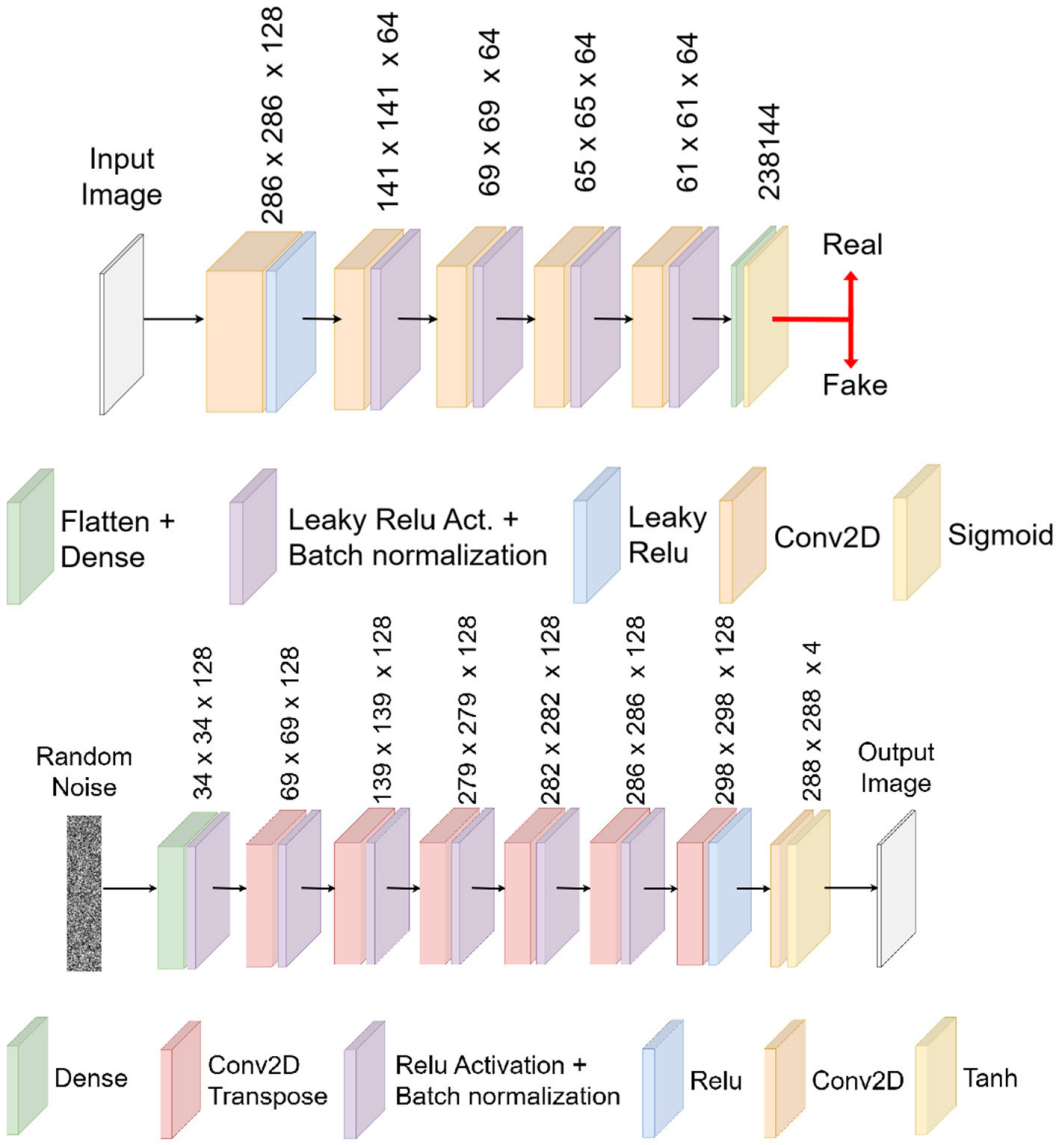
Discriminator architecture for the generative adversarial network **(upper image)** and generator architecture for the generative adversarial network **(lower image)**.

After testing different architectures, we found that the DCGAN model provided the most consistent and reliable results for our task. Its performance was superior in generating realistic synthetic data that effectively augmented the training set for our neural networks. Figures 4, 5 illustrate the specific architectures of the discriminator and generator used in this DCGAN model.

Note that the proposed DCGAN model was adapted to operate with (288 × 288 × 4) data structures. These structures are formulated through the concatenation of an RGB image, which signifies the input image, and a monochromatic image denoting the target image. In preparation for subsequent training of the detection neural model, it is imperative to disaggregate this composite data structure into its constituent components: an input RGB image with three color layers and a corresponding target image featuring a singular layer.

**Figure 6:**
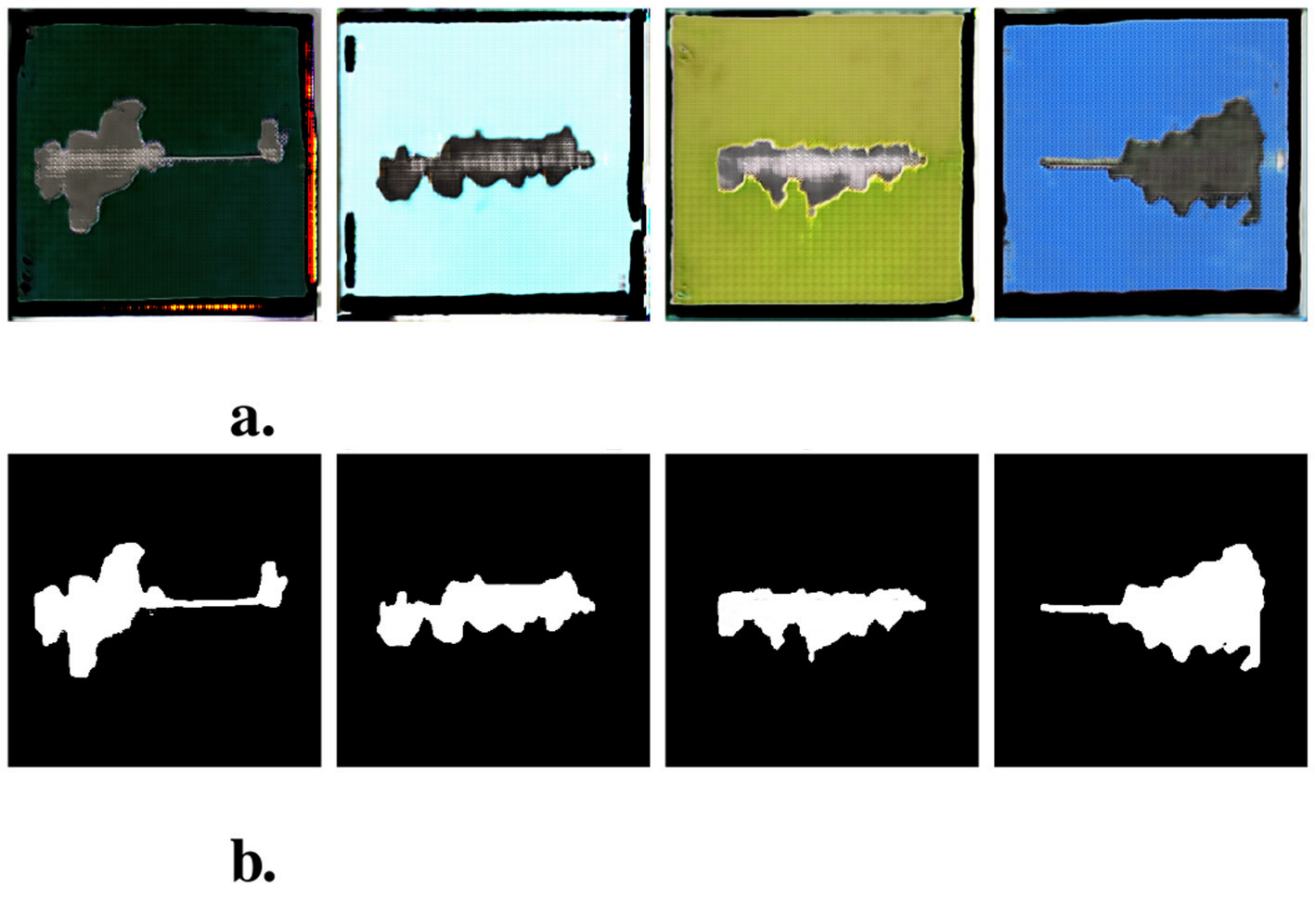
Examples of generated sythetic data. Each image in second row corresponds to image in first row. **(A)** Synthetic input images. **(B)** Synthetic target (mask) images.

A selected set of examples of synthetic data provided by this specific DCGAN model is depicted in Figure 6.

### 3.5 Variants of extended dataset

The crucial objective of this study is to systematically investigate and determine the optimal ratio between original and artificially generated data within the training dataset for the subsequent training of U-net and DeepLabV3 models, aiming to enhance the performance of the neural network models for the specific task of coating degradation detection. To achieve this, eight distinct training datasets were meticulously curated, each characterized by varying proportions of original and artificially generated data. These datasets will serve as the foundation for training the U-net and DeepLabV3 models, allowing for a comprehensive exploration of the impact of different ratios on the models' capacity to effectively detect and delineate coating degradation. The information about every variant of the training set is summarized in Table 1.

**Table 1:** Datasets with different ratios of original and synthetic data for detection model training.

**Original samples**	**Synthetic samples**	**Ratio Synthetic:Original**	**Label**
476	0	0	Ratio0.0
476	95	0.2	Ratio0.2
476	238	0.5	Ratio0.5
476	476	1	Ratio1.0
476	952	2	Ratio2.0
476	1,428	3	Ratio3.0
476	2,380	5	Ratio5.0
476	4,760	10	Ratio10.0

### 3.6 Evaluation metrics

To convincingly illustrate the advantages of automated dataset extension, a comprehensive set of evaluation metrics must be employed. A primary metric for assessing classification performance involves the calculation of accuracy over an independent test set. In the context of evaluating the degree of degradation, the task essentially entails pixel-wise classification of the image content.

For the classification of the degradation area, a true positive pixel is one labeled as white in both the target and the output images. Conversely, a false positive pixel is labeled as white in the output but as black in the target image. Similarly, a true negative pixel is one labeled as black in both the target and output images, while a false negative pixel is labeled as black in the output image but as white in the target image. The accuracy is then defined as


(3) $$\text{Accuracy}=\frac{\left|\text{TP}\right|\left|\text{TN}\right|}{\left|\text{TP}\right|+\left|\text{FP}\right|+\left|\text{TN}\right|+\left|\text{FN}\right|},$$

where TP, FN, FP, and TN represent the number of true positive, false negative, false positive, and true negative pixels in the tested sample, respectively.

To provide a comprehensive evaluation of the semantic segmentation performance, additional metrics are considered:


(4) $$\text{Precision}=\frac{\left|\text{TP}\right|}{\left|\text{TP}\right|+\left|\text{FP}\right|},$$

(5) $$\text{Recall}=\frac{\left|\text{TP}\right|}{\left|\text{TP}\right|+\left|\text{FN}\right|},$$

(6) $$\begin{array}{l} \text{F1-score}=\frac{2}{{\text{Recall}}^{-1}+{\text{Precision}}^{-1}}. \end{array}$$

Apart from accuracy, the F1-score emerges as the most informative metric among the evaluated performance measures. The F1-score, calculated as the harmonic mean of precision and recall, serves as a balanced indicator that considers both false positives and false negatives in the classification task. This balance is particularly crucial in tasks such as the pixel-wise classification of degradation areas, where achieving a harmonious equilibrium between correctly identifying positive instances (true positives) and minimizing the misclassification of negatives (false negatives) and positives (false positives) is paramount.

In order to directly reflect the practical applicability of the method, the ISO 4628-8 standard, described in Section 3.1, is also used to calculate the average width of the degradation area around the scribe for each sample, expressed in millimeters. Specifically, we will observe the difference in the width of degradation provided by developed model and by manual determination. This metric better reflects the real-world performance of the method in detecting degradation and provides a more tangible measure of the results.

## 4 Experiments and results

### 4.1 Setup of experiments

All experimental models were implemented using Python 3.9 with TensorFlow 2.0 and the Keras framework. Experiments were performed using the following hardware specification: Intel Core i9-14900K (3.2 GHz, BOOST 6 GB) processor, 64 GB DDR4 (2666 MHz) internal memory, NVIDIA GeForce RTX 4090 24 GB GDDR6X (16 384 CUDA cores) video card, SATA M.2 2,048 GB SSD.

### 4.2 Original dataset extension

To extend the original dataset, a GAN model was configured following the procedural framework illustrated in Figure 4. The training of the generator-discriminator followed the scenario provided in Linder-Norén (2019), with the only modification being a number of epochs, which now equals 500.

Subsequently, the trained GAN generator was leveraged to enhance the original dataset. Specifically, 4,760 synthetic input-target pairs with accordance to Table 1 were prepared using randomly generated input signals. Four illustrative examples from the generated dataset are presented in Figure 6.

### 4.3 Semantic segmentation model training

The training regimen for the detection of coating degradation encompassed three distinct models: U-net, DeepLabV3 with an Xception backbone, and DeepLabV3 with a MobileNetV2 backbone. Each of these models underwent training using eight distinct datasets, as delineated in Table 1. Notably, the models were trained utilizing the Adam optimizer, starting from scratch. The test set retained its original composition, while the training sets were extended with additional synthetic input-target pairs, resulting in a combined set of 24 training experiments. In addition, 15% of the training dataset values were allocated for use as a validation set. This partitioning strategy facilitated ongoing model assessment during training, allowing for the monitoring of performance on data not explicitly used for training.

Each training session was executed ten times in consideration of the stochastic nature inherent in the training process. This repeated training approach aimed to mitigate the effects of randomness and variability, ensuring a robust exploration of the model's parameter space. The iterative nature of the training procedure allowed for a comprehensive evaluation, enabling the models to converge to stable configurations and yielding more reliable and reproducible results.

The training hyperparameters adhered to the values specified in Table 2 and were kept consistent across all models to allow for a fair comparison between different dataset configurations. A sophisticated hyperparameter tuning process was not applied in this study. Instead, we chose standard and widely accepted settings to provide a baseline for evaluating the impact of dataset augmentation using synthetic data.

**Table 2:** Parameters of the training.

**Input shape**	**288 × 288 × 3**
Training algorithm	Adam optimizer
Loss function	Binary cross entropy
Number of training experiments for each session	10
Number of samples	456
Validation split	0.15
Initialization	Normal distrib. (mean = 0, std = 0.05)
Number of epochs	300
Batch size	16
Criterion for resultant model	Loss function value over validation set
Learning rate α	0.001
Exponential decay rate 1 β_1_	0.9
Exponential decay rate 2 β_2_	0.999

Hyperparameter tuning for each combination of dataset and model would have introduced an additional element of stochasticity, potentially distorting the results. By maintaining fixed hyperparameters, we aimed to minimize this effect, ensuring that any observed improvements or differences in model performance were directly attributable to the dataset augmentation rather than variability in hyperparameter selection.

The ensuing tables present the outcomes of the training process, providing a comparative analysis of the models' performance when trained with various extended datasets. The process encompasses all three models—U-net, DeepLabV3 with Xception, and DeepLabV3 with MobileNetV2—providing insights into how dataset extension influences their individual capacities for detecting coating degradation. The results, including metric values, are comprehensively summarized in Table 3.

**Table 3:** Resulting values of the considered metrics over the test set.

**Dataset**	**Accuracy**	**Precision**	**Recall**	**F1-score**
**U-net**				
Ratio0.0	0.9939	0.9360	0.9431	**0.9395**
Ratio0.2	**0.9940**	0.9427	0.9363	**0.9395**
Ratio0.5	0.9938	0.9291	0.9444	0.9367
Ratio1.0	0.9938	0.9257	0.9487	0.9371
Ratio2.0	0.9938	0.9219	0.9523	0.9369
Ratio3.0	0.9939	0.9282	0.9471	0.9376
Ratio5.0	0.9939	0.9352	0.9399	0.9375
Ratio10.0	0.9938	0.9237	0.9504	0.9369
**DeepLabV3**+**Xception**				
Ratio0.0	0.9918	0.8808	0.9466	0.9125
Ratio0.2	**0.9925**	0.9093	0.9362	**0.9225**
Ratio0.5	0.9923	0.9204	0.9200	0.9202
Ratio1.0	0.9924	0.9219	0.9082	0.9150
Ratio2.0	0.9919	0.9081	0.9200	0.9140
Ratio3.0	0.9919	0.9212	0.9093	0.9152
Ratio5.0	0.9918	0.9046	0.9176	0.9110
Ratio10.0	0.9916	0.8924	0.9289	0.9103
**DeepLabV3**+**MobileNetV2**				
Ratio0.0	0.9822	0.8901	0.9373	0.9131
Ratio0.2	**0.9937**	0.9363	0.9255	0.9309
Ratio0.5	0.9936	0.9411	0.9305	**0.9358**
Ratio1.0	0.9933	0.9284	0.9313	0.9298
Ratio2.0	0.9933	0.9540	0.9045	0.9286
Ratio3.0	0.9936	0.9464	0.9162	0.9310
Ratio5.0	0.9934	0.9310	0.9275	0.9292
Ratio10.0	0.9934	0.9512	0.9132	0.9318

The highest values for the most important metrics (Accuracy, F1-score) are highlighted.

**Figure 7:**
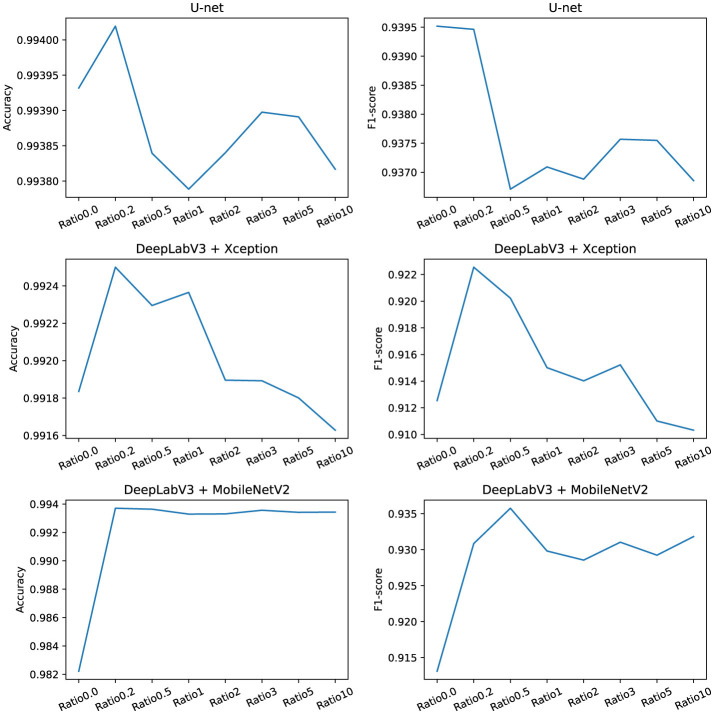
Courses of selected metrics.

For a more illustrative representation of the values, the two most important metrics, namely accuracy, and F1-score, were graphically depicted in Figure 7.

Furthermore, the ISO 4628-8 standard was evaluated on all trained models across all samples from the test set. The table presents the number of cases where the model provided the correct degradation width, as well as instances where the error was within ±1 mm, within ±2 mm, and cases where the error exceeded ±2 mm. The resulting values are shown in Table 4.

**Table 4:** Resulting differences in values of the ISO 4628-8 standard over the test set.

**Dataset**	**0 mm**	**±1 mm**	**±2 mm**	**>±2 mm**
**U-net**				
Ratio0.0	118	8	0	2
Ratio0.2	**120**	6	0	2
Ratio0.5	116	10	0	2
Ratio1.0	116	10	0	2
Ratio2.0	112	13	2	1
Ratio3.0	117	8	1	2
Ratio5.0	118	6	1	2
Ratio10.0	114	11	1	2
**DeepLabV3**+**Xception**				
Ratio0.0	97	28	2	1
Ratio0.2	**113**	12	1	2
Ratio0.5	**113**	13	0	2
Ratio1.0	**113**	13	0	2
Ratio2.0	103	22	1	2
Ratio3.0	107	19	0	2
Ratio5.0	105	19	2	2
Ratio10.0	96	30	1	1
**DeepLabV3**+**MobileNetV2**				
Ratio0.0	111	11	3	3
Ratio0.2	**117**	09	0	2
Ratio0.5	116	10	0	2
Ratio1.0	**117**	9	0	2
Ratio2.0	112	14	0	2
Ratio3.0	114	12	0	2
Ratio5.0	114	11	2	2
Ratio10.0	111	15	0	2

Bold values indicate the highest number of samples evaluated with zero error.

### 4.4 Discussion

The experimental results show that the use of synthetic data improves the performance of both U-net and DeepLabV3 models, as measured by accuracy and F1-score metrics, as well as by the final values provided by the assessment method provided within the ISO 4628-8 standard. The results also reveal that different models may have different preferences for the ratio of synthetic to original data. However, the optimal ratio always tends to values between 0.2 and 0.5. The higher synthetic data ratio especially in U-net and DeepLabV3 architecture with Xception backbone leads to a sharp drop in F1-score. These findings suggest that the quantity of artificially generated data must not surpass the quantity of original data; instead, the ratio should approach half of the original dataset.

U-net, which has a symmetrical and densely connected structure, may benefit from a lower ratio of synthetic data, as it can effectively capture and localize the features of the original data. DeepLabV3, which has an asymmetrical and sparsely connected structure, may profit from a higher ratio of synthetic data, as it can leverage the multi-scale and diverse information provided by the synthetic data. Moreover, the choice of the backbone network may also influence the preference for the ratio of synthetic to original data. Xception, which has a deeper and more powerful structure, may require more balanced and varied data to avoid overfitting and underfitting. MobileNetV2, which has a shallower and more efficient structure, may require more synthetic data to compensate for the loss of information and expressiveness.

The use of GANs for dataset augmentation has been widely explored in the domain of medical imaging, where the scarcity of original labeled data presents a similar challenge to the one encountered in our study. In medical imaging, GAN-generated data has been shown to improve model performance significantly, with accuracy increases ranging from a few percentage points, as seen in Tekchandani et al. (2020), to as much as 14% presented in Wang et al. (2019). These studies demonstrate the effectiveness of GAN-based augmentation in addressing the problem of limited data. However, direct comparison with our method is difficult due to the highly specific nature of our dataset. While medical images often involve uniform imaging conditions and clearly defined anatomical structures, the surface textures and degradation patterns in our coil-coated samples are much more diverse. Thus, the gains seen in medical imaging may not fully translate to the domain of surface degradation. However, both domains indicate the positive benefit of using GANs for dataset augmentation.

Considering dataset augmentation for coating degradation detection, the approach described by Tao et al. (2024) relies on the input of a predefined degradation mask and outputs only the reconstructed original image. While this approach offers precise control over the generation of synthetic original images based on provided masks, our method is more general in that it does not require prior knowledge of the degradation patterns. This allows for the generation of entirely new synthetic samples in a single automatic step. However, one limitation of our approach is that it does not allow for direct control over the distribution of different degradation levels within the synthetic dataset. In some cases, this could lead to an imbalance in the representation of various degradation stages. As a result, further processing of the augmented dataset may be required to ensure an even distribution of degradation levels across the dataset, thereby enhancing the model's ability to generalize across all degrees of degradation.

The study also provides insights into the strengths and limitations of the DCGAN model for generating synthetic data. The visual evaluation of the synthetic data shows that the DCGAN model can produce realistic and diverse images of coil-coated surfaces with different colors, textures, and reflectivity. The synthetic data also exhibit various degrees of degradation around the scribe, which are consistent with the original data. However, the DCGAN model also has some drawbacks, such as producing artifacts, blurring, and distortion in some images, which may affect the quality and fidelity of the synthetic data.

### 4.5 Future directions

The study opens up several avenues for future research and improvement. One important direction is to further refine the architecture and training process of the GAN model. As observed in the experiments, the ratio of synthetic to original data significantly affects the performance of the model, and controlling the type of generated data in terms of degradation severity and material properties could lead to better results. This could be achieved by incorporating additional constraints or objectives into the GAN training process, or by employing conditional GANs, which allow for the generation of data based on specific input conditions or labels. In particular, the use of conditional GANs could enable more precise control over the distribution of degradation levels, addressing the current limitation of our method, where varying degrees of degradation are not explicitly regulated. Ensuring a more even representation of different degradation stages could further enhance the model's ability to generalize across diverse real-world conditions.

Another promising avenue for future research is to investigate more advanced techniques for optimizing the GAN architecture, such as reinforcement learning or meta-learning. These techniques could be used to automatically adjust the parameters and architectures of both the GAN and the segmentation models, based on their performance metrics. This approach could lead to a more efficient and dynamic process for generating synthetic data that is tailored to specific application needs. Furthermore, research into optimizing the balance between synthetic and real data could provide insights into how to dynamically adjust this ratio during training to maximize model performance without overfitting or underfitting.

Additionally, the proposed methodology could be adapted and applied to other domains where semantic segmentation is critical. For example, the use of GANs for dataset augmentation has been extensively studied in medical imaging, as mentioned in the Discussion section, and applying our method to this field could provide valuable insights. Other potential applications include autonomous driving, where diverse data are needed to handle various road conditions and object appearances, and remote sensing, where generating synthetic satellite or aerial imagery could enhance the performance of models trained for land use classification or environmental monitoring. Exploring the adaptability of our GAN-based approach in these contexts could reveal its broader applicability and help address challenges in other fields requiring robust data augmentation solutions.

## 5 Conclusions

The main contribution of this study is the introduction of a GAN-based methodology for extending the training dataset for semantic segmentation tasks, specifically aimed at detecting the degree of coating degradation around a scribe. By employing a Deep Convolutional GAN, our approach effectively generates synthetic input-target pairs, both original images and corresponding degradation masks. This dual generation process significantly simplifies the task of dataset augmentation, as it eliminates the need for manual annotation of synthetic images, which is typically required when using other augmentation techniques. The generated synthetic data accurately mimics the variability in real-world samples, including diverse colors, textures, and surface reflectivity, which are characteristic of coil-coated surfaces.

The results of this study demonstrate that augmenting the original dataset with synthetic data improves the performance of two state-of-the-art semantic segmentation models, U-net, and DeepLabV3. The effectiveness of the GAN-augmented dataset is evident across multiple evaluation metrics, including accuracy and F1-score. Furthermore, the study reveals that the optimal ratio of synthetic to original data falls between 0.2 and 0.5, depending on the model architecture. In particular, U-net benefits from a lower ratio of synthetic data, while DeepLabV3, especially with the Xception backbone, can utilize a higher ratio, up to a point, before performance begins to degrade.

This study highlights the potential of GAN-based dataset augmentation to address the challenges posed by limited annotated data, particularly in industrial applications where data collection and labeling can be resource-intensive.

While our method shows promising results, there are areas for further improvement, including the possibility of controlling the degree of degradation in the generated data and ensuring a balanced distribution of degradation levels across the dataset. Despite these challenges, the approach outlined in this study offers a scalable and effective solution for augmenting datasets, with the potential for application in a wide range of semantic segmentation tasks beyond surface degradation detection.

## Data Availability

The raw data supporting the conclusions of this article will be made available by the authors, without undue reservation.
